# (Bi,Sr) (Fe_1−*x*_,M*_x_*)O_3−δ_ (M = Co, Ni and Mn) Cathode Materials with Mixed Electro-Ionic Conductivity

**DOI:** 10.3390/ma9110922

**Published:** 2016-11-14

**Authors:** Wen-Cheng J. Wei, Der-Rong Huang, Dan Wang

**Affiliations:** Department of Materials Science and Engineering, National Taiwan University, Taipei 106, Taiwan; r00527050@ntu.edu.tw (D.-R.H.); wendan0505321@gmail.com (D.W.)

**Keywords:** bismuth ferrite, dopant, cathode, fuel cell, ionic, conductivity

## Abstract

(Bi,Sr)FeO_3−δ_ (BSF) cathode materials doped with either Co, Ni or Mn are synthesized by an ethylene diamine tetra-acetic acid (EDTA)-citrate complexing method, and the effects of the doping level on the mixed electronic-ionic conductivity at various temperatures are studied up to 800 °C. The phase purity and solid solution limit are investigated by X-ray diffraction (XRD). The ionic conductivity is measured by the four-probe direct current (DC) method, the valence state of Fe and Mn by X-ray photoelectron spectroscopy (XPS), and the oxygen non-stoichiometry by differential thermo-gravimetric analysis (TGA). The doped ferrites show interesting electronic conductivity dependent on the testing temperature, implying two conductive mechanisms, either controlled by double exchange at lower temperatures or small polaron (electron-oxygen vacancy) conduction at temperatures greater than 400 °C. The results of Co-doped BSF (S50C20) show the best mixed conductivity among the ferrites, and this is used to assemble cells. The cell with a S50C20 cathode in the region of 600–800 °C is improved by 15% in maximum power density greater than the cell with La_0.6_Sr_0.4_Co_0.2_Fe_0.8_O_3−δ_ (LSCF) due to the balanced contribution from oxygen ions, vacancies and electrons.

## 1. Introduction

Solid oxide fuel cells (SOFCs) are power generation devices that directly convert fuels into electricity via electrochemical reactions through clean and highly efficient operations. To improve the reliability and stability of the cells, lowering the operating temperature to an intermediate-temperature range (below 800 °C) [1,2] is one of the main targets in the recent research efforts. These advantages of lower-temperature operation, including the ease of material selection, reduction in total cost and accelerating the start-up process, are generally recognized. At present, the operation of SOFCs at 650 °C to 800 °C is currently feasible for a SOFC using ultrathin yttria-doped zirconia [3]. However, a lower operating temperature results in sluggish oxygen reduction reaction (ORR) at the cathode and apparently increases the resistance of the electrolyte/cathode, potentially degrading the performance of SOFCs [4,5].

High performance cathodes based on La-perovskite (LaMO_3_) materials have been studied, and it was recently reported that cobalt-containing (La,Sr)CoO_3_ perovskites exhibit the best activity of electro-catalytic ORR at intermediate temperatures (600 °C–800 °C). Since cobalt is a good catalyst for reduction oxygen molecules to ions and flexible redox behavior via valence transition [4], the cobalt containing perovskites, e.g., La_0.6_Sr_0.4_Fe_0.8_Co_0.2_O_3−δ_ (LSFC6482), have a good electronic conductivity, However, they have very low ionic conductivity and the high coefficient of thermal expansion (CTE) of the LSFC cathode mismatches to that of zirconia-based electrolyte, which is also unfavorable to thermal cycling of the cells [6,7,8,9,10]. Besides, an interfacial reaction might occur in various cathode/yttria-stabilized zirconia (YSZ) combinations. Amorphization of YSZ by the diffusion of the Mn from (La,Sr)MnO_3−δ_ cathode [11], or the formation of a new Co-phase by (La,Sr)(Fe,Co)O_3−δ_ [12] are reported in the literature.

A new cathode material based on (Bi,Sr)FeO_3_ (BSF) was synthesized and reported with interesting conductivity [13,14,15,16,17,18,19,20,21]. To enhance the conductivity and ORR capability, Co [18,21], Ca [19] and Mn [20] were doped in the BSF perovskite structure. Besides, a higher oxygen exchange rate of pore-free BSF was reported, even higher than that of (La,Sr)FeO_3−δ_. The literature [21] concludes that the BSF structure with large ionic sizes of Bi^3+^ and Sr^2+^ at A-site, and Fe^3+^ in B-site are basically performed favorable oxygen ion conductivity and matched CTE to the Y-doped ZrO_2_ electrolyte. The substitution of A and B sites in the perovskite structure also affect the oxygen stoichiometry. A controversial report on the low oxygen stoichiometry of (Bi,Sr)FeO_3−δ_ varying over the range of 0 < δ < 0.016 [19] and δ = 0.23 [15], is noted in the literature. Besides, the measurement method of the ionic conductivity, i.e., electrochemical polarization by electric impedance spectroscopy (EIS), was used in many papers and might give over-promising conductivity values (1.4 × 10^−2^–2.6 × 10^−1^ S·cm^−1^) of the BSF at 650 °C. The ionic conductivity of the ferrites with respect to oxygen deficiency needs careful verification.

A series of Sr-doped LaMnO_3_ cathode material likely enhances the electronic conductivity due to the charge exchange of Sr_La_ and Mn^n+^ ions in the high-temperature conditions of the stoichiometry composition [11,22]. The possible reaction is shown as below:

(1) $${\mathrm{Sr}}_{\mathrm{La}}+{\mathrm{Mn}}^{3+}↔{\mathrm{Sr}}_{\mathrm{La}}^{1-}+{\mathrm{Mn}}^{4+}\;\mathrm{or}\;{\mathrm{Sr}}_{\mathrm{La}}+{\mathrm{Mn}}^{2+}↔{\mathrm{Sr}}_{\mathrm{La}}^{1-}+{\mathrm{Mn}}^{3+}$$

This likely goes through the double-exchange of electrons between neighboring cations (M). In a more general form, the double-exchange conductive mechanism of moving electron between cations can also be shown as below when considering the presence of oxygen between the cations (M^+^):

(2) $$M^{n+}-O^{2-}-M^{(n+1)+}\to M^{n+}-O^{1-}-M^{n+}\to M^{(n+1)+}-O^{2-}-M^{n+}$$

An additional possible conductive mechanism, small polaron, was proposed by Tuller and Nowick [23], which was mainly controlled by the existence of sufficient oxygen vacancy concentration, ≥1%. The association of electron with point defects, e.g., oxygen vacancies, inducing rapid transport of electron between the vacancies is generally accepted due to a weak polarization interaction, and is used in the interpretation of oxide conductivity at high temperature [24,25].

This study uses a molecular-scale synthesis method (termed the “EDTA-citrate method”) to fabricate homogeneous (Bi_1−*x*_Sr*_x_*)(Fe_1−*y*_M*_y_*)O_3−δ_ powders, and low temperature sintering to obtain various porous cathode materials. Therefore, we can understand the conductive mechanisms of the cathode materials. The electric conductivity, including ionic and electronic, will be measured by the two-probe and four-probe ionic DC method [26]. The mixed-ionic-electric (MIE) property of the doped ferrites is tailored in this study to optimize the electrochemical properties of the cathodes and used to improve the performance of made fuel cells.

## 2. Results

### 2.1. BSF with Co-Doping

Two series of the ferrite are reported in this section. Their abbreviations, sintering properties and other properties are reported in Table 1. The synthesized S*x*-series and S*x*C*y*-series powders can be densified to a relative density better than 95% theoretical density (T.D.). Thermal expansion analysis was conducted from room temperature to 900 K (627 °C). The coefficient of thermal expansion (CTE) for S*x*-series is in the range of 10 × 10^−6^–15.1 × 10^−6^ K^−1^, and 15.4 × 10^−6^–19.3 × 10^−6^ K^−1^ for co-doped S*x*C*y* series.

**Figure 1:**
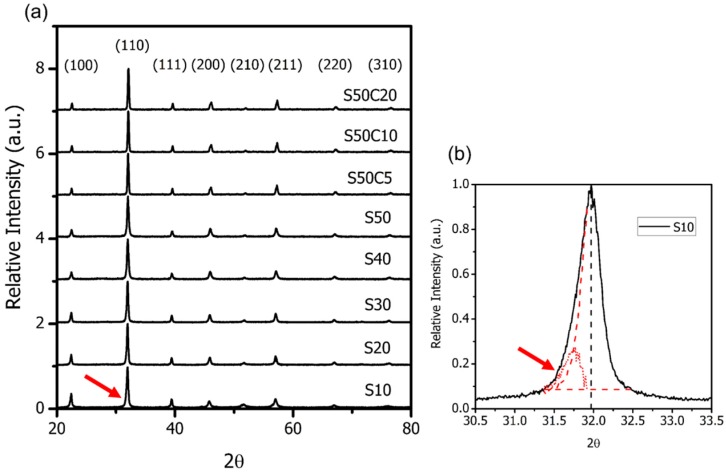
XRD patterns of bismuth ferrites with different Sr-doping and Co-doping contents in the range of 2θ: (**a**) from 20° to 80°; and (**b**) from 30.5° to 33.5°. Note the (110)_c_ peak of S10 sample after de-convoluted to two. The smaller one may belong to (104)_r_ of a rhombohedral structure.

The X-ray diffraction (XRD) results of the powders with different SrO and CoO-doping contents calcined at 750 °C for 10 h are shown in Figure 1. Most of the BSFM*x* display a pure perovskite (a symmetry of Pm-3m), except the S10 sample, which shows a (110)_c_ peak with a very minor (104)_r_ intensity belonging to a rhombohedral structure in a symmetry of R3C. No other secondary phases are found over the composition range. The evidence reveals the substitution of Bi^3+^ by Sr^2+^ and Fe^3+^ by Co^2+^ are complete, resulting in the formation of an oxygen vacancy and multiple valence states at the Fe-site. These will be discussed in Section 3.

**Figure 2:**
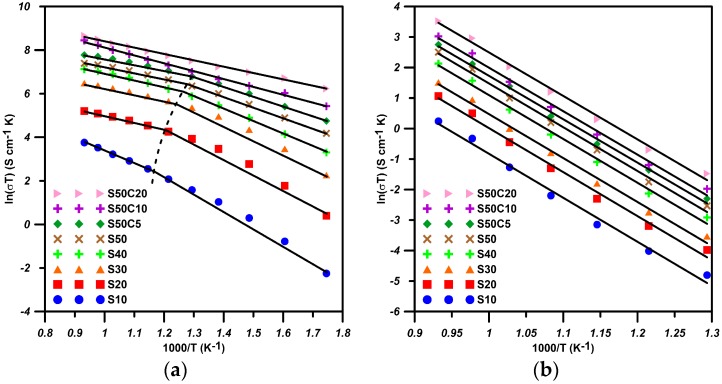
Arrhenius plot of: (**a**) electrical conductivity; and (**b**) ionic conductivity of S*x*C*y* bismuth ferrites with different Sr- and Co-doping.

The ferrite samples were densified to a relative density better than 95.0% T.D. (Table 1), and then put to conductivity measurement. The total conductivities of Bi_1−*x*_Sr*_x_*FeO_3_ and Bi_0.5_Sr_0.5_Co*_y_*Fe_1−*y*_O_3_ are shown in Figure 2. The data depict that the electrical conductivity increases with increasing temperature. The S50 sample has the best electrical conductivity among the Sr-doped bismuth ferrites, reaching a maximum value of 1.5 S·cm^−1^ at 800 °C. In the S50C*y* ferrites co-doped with cobalt, S50C20 shows the best electrical conductivity of 5.4 S·cm^−1^ at 800 °C.

The temperature dependence of the electric conductivity of the ferrites is noted changing at about 500 °C–527 °C, as shown by the dot curve in Figure 2a. The slopes of the best fitting lines are calculated to reveal the activation energy of the conductivity. The energies and transition temperatures of all S*x*C*y* are shown in Table 2. There are no such transitions in the S50C10 and S50C20 samples. The *E*_a_(LT) of S*x* samples is in the range of 0.4 eV to 0.7 eV, but *E*_a_(HT) is in the range of 0.21 eV to 0.48 eV. The SrO-doped BSFs possibly act as a double exchange mechanism at a lower temperature range, and change to a vacancy control mechanism as the temperature goes higher than *T*_tr_. For Co-doped cases, the electronic conduction in S50C10 and S50C20 still retains the double exchange mechanism up to 800 °C. Later, XPS/TGA results will be provided to discuss the possible conductive mechanisms.

Figure 2b shows the temperature dependence of the ionic conductivity of all S*x*C*y* ferrites. The ionic conductivity increases with testing temperature, and displays a single slope through the testing temperature range. The correspondent activation energies show a similar value of 1.24 ± 0.06 eV for all S*x*C*y* ferrites, lower than that of 1.32–1.34 eV for LSCF6428 [11]. In the series of Bi_1−*x*_Sr*_x_*FeO_3_, the best ionic conductivity is 0.032 S·cm^−1^ among all samples at 800 °C which is apparently higher than that (2.2 × 10^−^^3^–4 × 10^−3^ S·cm^−1^) of LSCF82*xx* at the same temperature [11].

**Figure 3:**
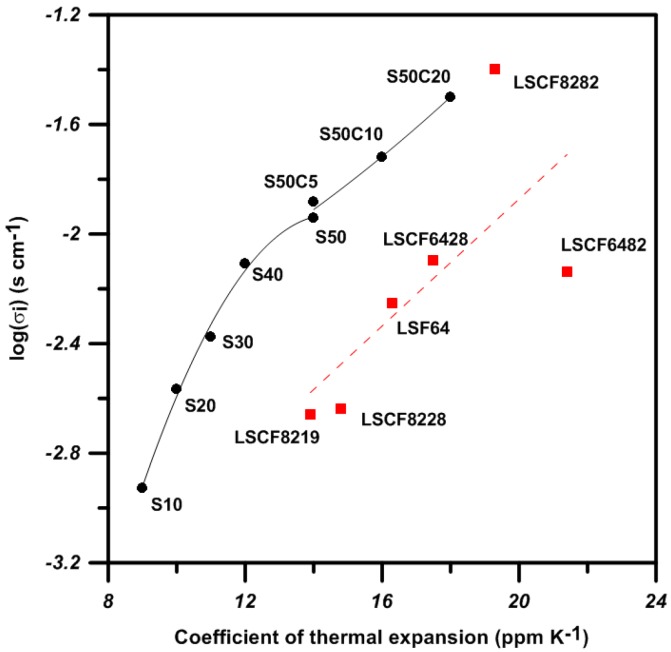
Ionic conductivity at 800 °C of (Bi,Sr)FeO_3_ and (Bi,Sr)(CoFe)O_3_ plotted against CTE (room to 800 °C) and the data (dot line) of La-perovskites [11]. Note the numbers following LSCF are the proportion of the La, Sr, Co, Fe elements.

The ionic conductivity and the CTE of S*x*C*y* shown in Table 1 and Figure 2b are plotted and compared with that of LSCF in Figure 3. The σ_i_ values of the Bi-based perovskites are in general greater than that of the La-perovskites. Besides, the Bi-ferrites show two-stage increment of ionic conductivity. The first is increased by Sr-doping, and the second is by additional Co-doping. Based on the CTE match to that of yttria-doped ZO_2_, three S30M*y* series (M = Co, Ni and Mn) possibly showing a CTE of ca. 12 × 10^−6^ K^−1^ were selected for further investigation.

### 2.2. S30 with Co, Ni or Mn Doping

**Figure 4:**
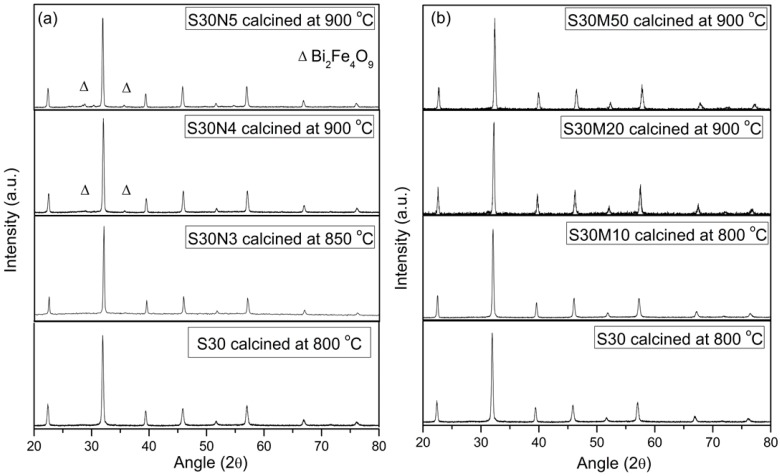
XRD patterns of: (**a**) Bi_0.7_Sr_0.3_Fe_1−*x*_Ni*_x_*O_3_ (S30N*x*) with the impurity phase Bi_2_Fe_4_O_9_ powder (PDF#20-836); and (**b**) Bi_0.7_Sr_0.3_Fe_1−*x*_Mn*_x_*O_3_ (S30M*x*) calcined at 800–900 °C.

The solubility of Co, Ni and Mn performs differently in Bi_0.7_Sr_0.3_FeO_3_ (S30). A previous report by Chen [27] showed that only 4% of Co can form a solid solution in S30C*y* ferrite as the Sr-content was reduced to 30% (i.e., S30C4). The same testing procedure of Ni and Mn was conducted and determined by XRD, as shown in Figure 4. After calcining at 900 °C, a secondary phase (the impurity phase) Bi_2_Fe_4_O_9_ was found in Ni-doped BSFs, i.e., S30N4 and S30N5, indicating that a 4 at % doping level of Ni was over the solubility limit of the S30N*y*. Therefore, the maximum Ni soluble content in BSF was 3 at %. Similar to previous procedure, Mn was also doping in BSF, 50 at % Mn-doped S30 still appeared in a single perovskite phase (Figure 4b). The result is similar to that reported by Baek et al. [20].

**Figure 5:**
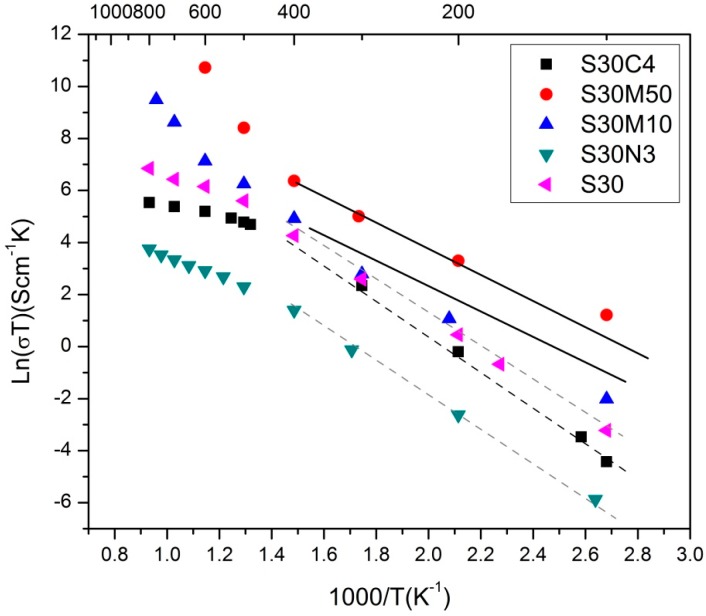
Arrhenius plot of electric conductivity of doped S30 as a function of the reverse of the testing temperature (1/*T*). The S30C4 results reported by Chen [27] are plotted in the diagram.

The electrical conductivity of the S30M*y* is shown in Figure 5. The samples were sintered at 1000 °C for 4 h, and achieved 92.5%, 91.6% and 95.1% relative density (R.D.) for S30, S30N3 and S30M50, respectively. The S30 data in the Figure appear to be well matched to that of the same composition reported by Niu et al. [15]. The electrical conductivity of their (Bi_0.7_Sr_0.3_)FeO_3−δ_ at 800 °C was 1.2 S·cm^−1^, which was almost the same as our data.

All samples in Figure 5 showed a single perovskite phase. The conductivity of three doped S30 series displayed similar conductive behavior. The temperature dependence of the electric conductivity of all ferrites appeared two activation energy levels (*E*_a_) either at low or high temperature separated by a transition temperature (*T*_tr_), as summarized in Table 3. The samples had approximately two *E*_a_(LT) values in the lower temperature region, either 0.55 ± 0.04 eV or 0.40 ± 0.03 eV (Table 3). The conduction behavior was possibly controlled by a double-exchange mechanism (M*^n^*^+^-O^2−^M^(*n*+1)+^) along the Fe(M)O_6_ octahedral chains in the perovskite structure.

As the temperature increased to be greater than *T*_tr_, the *E*_a_(HT) either decreased to 0.25–0.36 eV or increased to 1.09 eV·mol^−1^. The *E*_a_(HT) of three samples, S30, S30N3 and S30C4, at lower range possibly indicated that the oxygen site of the double-exchange mechanism replaced by the oxygen vacancy (M-V_O_-M), therefore, the other mechanism dominated. A small polaron ($e^{-}{:V}_{O}^{2+}$) offered conductivity through the exchange of electrons by the neighboring oxygen vacancies. On the other hand, the apparent increase in the conductivity of S30M10 and S30M50 at higher temperature was noted, showing the highest *E*_a_(HT) among the Bi-ferrites. This might be caused by the multiple valence states of Mn and/or by the introduction of more oxygen vacancies than BSF and BSFN03. The evidence from the XPA and TGA results in the following Section 2.3 and Section 2.4 being presented to support the interpretations.

### 2.3. Valence State Analysis

**Figure A1:**
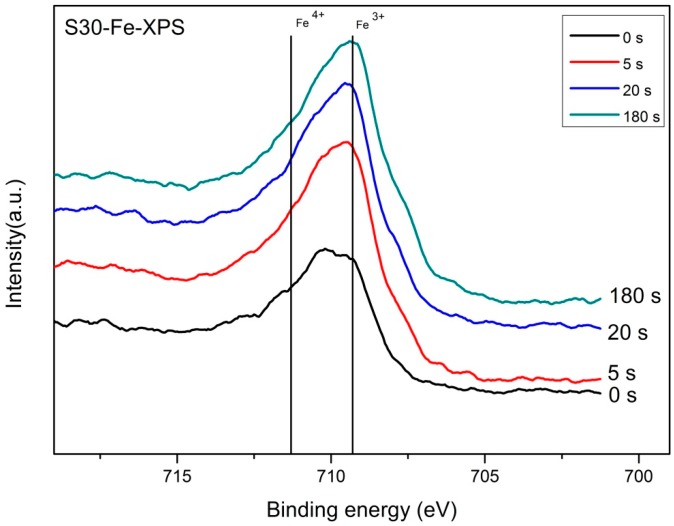
XPS Fe 2p_3/2_ spectra of the S30 surface been pre-etched by Ar^+^ for specified period, where 0 s means no Ar^+^ etching.

To investigate the effects of different Ar^+^ sputtering periods (0–180 s) on the valence states of Fe in S30 were first investigated and the spectra are shown in Figure A1. The binding energy of Fe on the B-site of perovskite and the double perovskite structure have been reported and summarized in previous references [28,29,30,31,32]. *E*_b_ of Fe^3+^ 2p_3/2_ was around 709.5 eV–710.2 eV, while the *E*_b_ of Fe^4+^ 2p_3/2_ was from 711.2 eV to 712.8 eV. The peak in Figure A1 appearing at 709 eV–713 eV contained more than one emission, indicating the possible co-existence of Fe^3+^ and Fe^4+^. No Fe^2+^ signals in the range of 708.8–710.8 eV were indexed [33].

**Figure A2:**
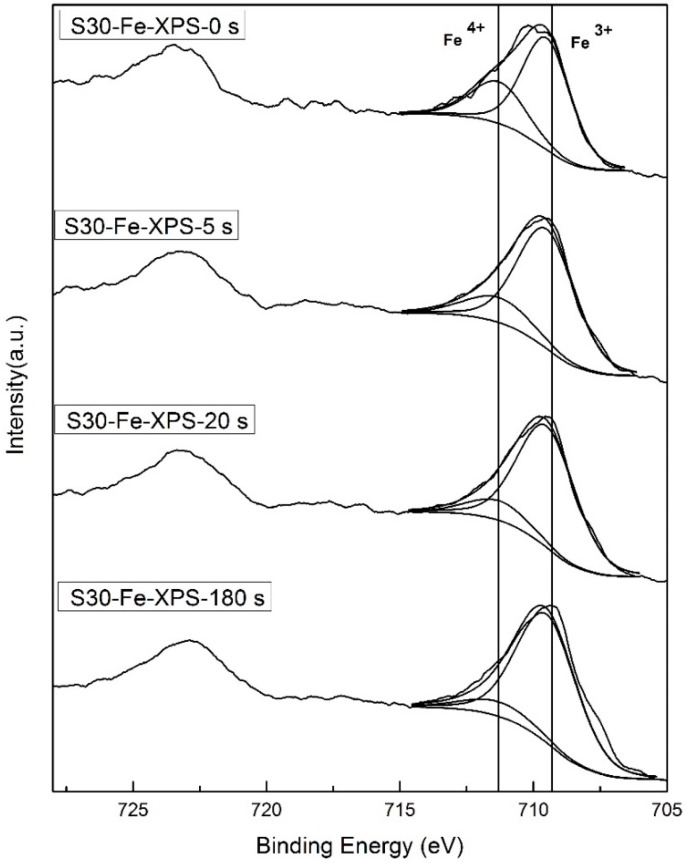
XPS spectra of Fe 2p_3/2_ of the polished surface of S30 after Ar^+^ been etched for 0 s, 5 s, 20 s, and 180 s.

Note that the Fe 2p_3/2_ peak was significantly shifting to a lower binding energy after sputtering for 180 s. According to the report [34], both Fe_3_O_4_ and Fe*_x_*O samples of newly made and stabilized in air were covered with a thin layer of Fe_2_O_3_, The Ar^+^ bombardment could remove the covered oxide layer in 5 s and reveal the original states within the samples. Similarly, when the samples were exposed to air, the proportion of oxygen states changed, as shown in Figure A2. According to the electric neutrality, the valence state of Fe would increase. Thus, a part of Fe^3+^ on BSF surface would oxidize to Fe^4+^. After slightly sputtering for 5 s at an acceleration voltage of 3 keV, the oxidized layer on BSF surface was removed.

In this study, the samples showed similar Fe 2p_3/2_ spectra after etching for 5 s, 20 s and even for as long as 80 s. For a longer period than or equal to 180 s, the Fe 2p_3/2_ peak shifted to a lower binding energy and the spectra indicated a reduction of Fe^4+^ to Fe^3+^. Thus, it is concluded that a limited sputtering (3 keV for 5 s) can remove the surface oxide layer on the BSF surface. Therefore, all samples in the following XPS study will go through sputtering for 5 s before the analysis.

**Figure 6:**
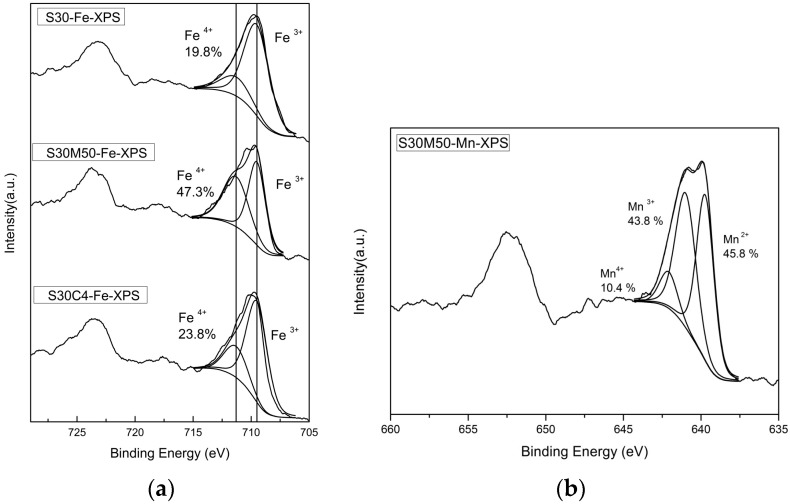
XPS spectra of: (**a**) Fe 2p_3/2_ of the polished surfaces of three S30, S30M50 and S30C4 samples; and (**b**) Mn 2p of the polished surface of the S30Mn50 after Ar^+^ etching for 5 s.

Figure 6a shows the XPS spectra of Fe 2p_3/2_ of the polished surfaces of three samples, S30, S30M50 and S30C4, after Ar^+^ etching for 5 s. the content of Fe^4+^ of these are 19.8%, 47.3% and 23.8%. The average valence state of Fe by calculation is 3.20, 3.47 and 3.42, respectively. Co and Mn doping all increase the Fe^4+^ content in the doped S30 samples.

The XPS spectra of Mn 2p of the polished BSFMn0.5 surface following Ar^+^ etching for 5 s are shown in Figure 6b. Mn^2+^, Mn^3+^ and Mn^4+^ were indexed in the spectrum of BSFMn0.5. The average valence state of Mn ions in BSFMn0.5 is 2.65. These results are similar to the work reported by Chuang et al. [35] and the presence of Mn^2+^ increased the oxygen vacancy concentration. As the valence state of Bi and Sr in A-site are constant, i.e., trivalent and divalent, respectively, the oxygen non-stoichiometry is mainly controlled by the valence state of the cations in the B-site. Thus, the oxygen stoichiometry of the S30, S30M50 and S30C4 equilibrium in the atmosphere and at room temperature are 2.95, 2.88, and 2.97, respectively. The S30M50 shows the greatest non-stoichiometry (Δδ = 0.12) of oxygen among the samples at room temperature.

**Figure 7:**
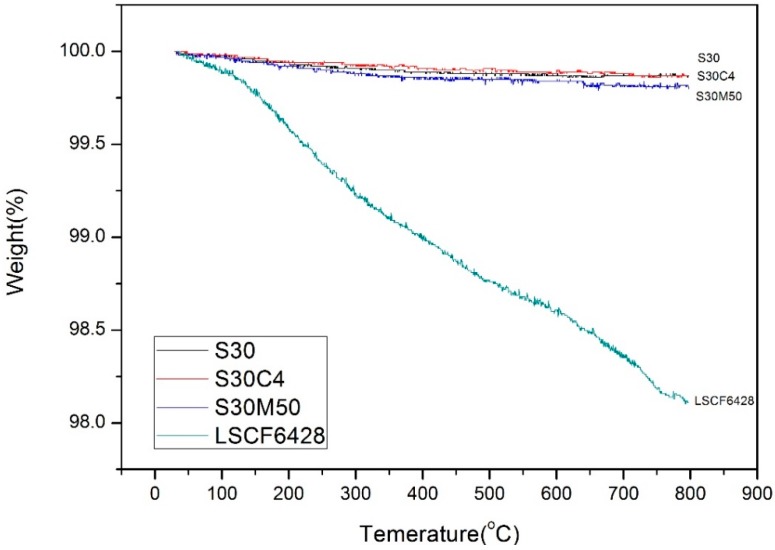
TGA curves of the S30, S30C4, S30M50 and LSCF6428 tested in air.

**Figure A3:**
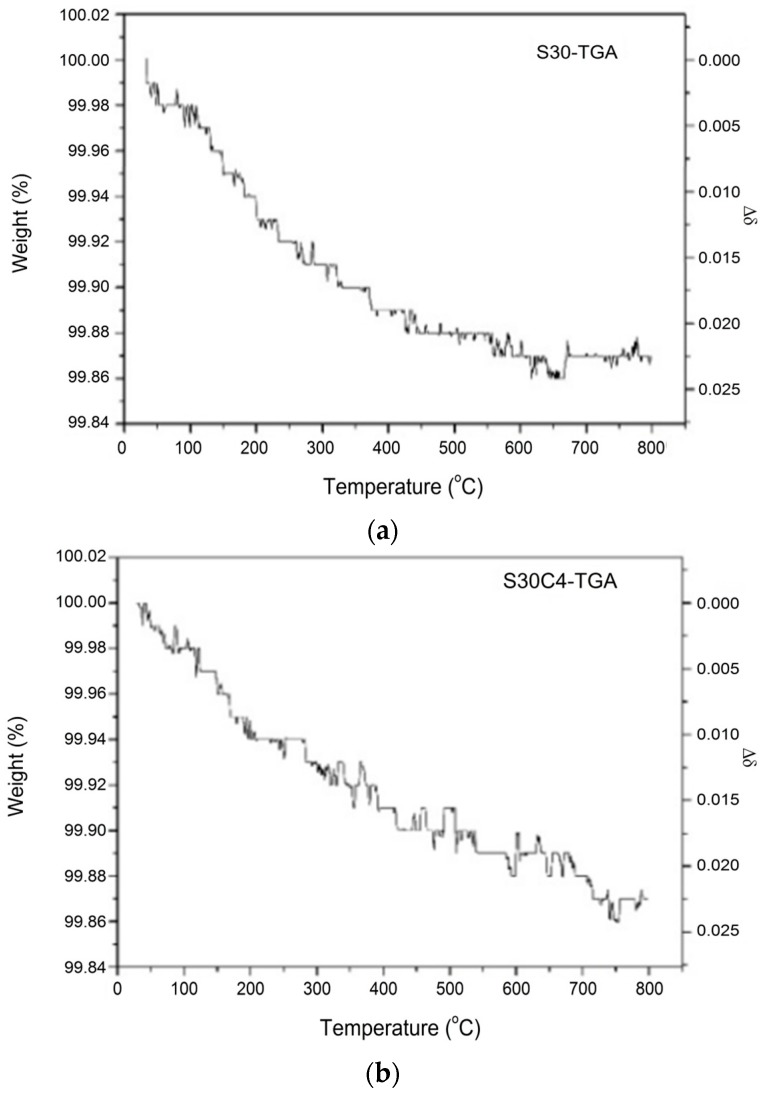

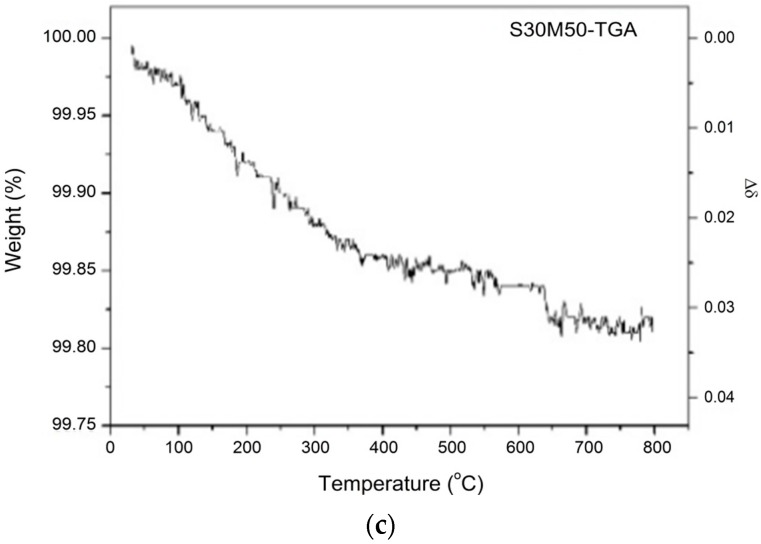
TGA and oxygen deficient (Δδ) curve of: (**a**) S30; (**b**) S30C4; and (**c**) S30M50 measured from room temperature to 800 °C.

The oxygen non-stoichiometry (Δδ) of the sample while undergoing the thermo-gravimetric analysis (TGA) test to high temperature was calculated from the mass loss, as shown below:

(3) $$\Delta \delta =\;\frac{M_{s}}{m_{s}M_{O}}\Delta m$$

where *M*_s_ is the molar mass of the stoichiometric oxide (g·mol^−1^) of BSFM*x*, *m*_s_ is the sample mass of the stoichiometric oxide of BSFM*x*, and *M*_O_ is the molar mass of oxygen (g·mol^−1^), and Δ*m* is the measured mass loss in the sample. We assume that the sample will not lose any of the cations (not vaporizing), but the oxygen may release and create a new oxygen vacancy when it is heated to 800 °C. The TGA curves of S30, S30C4 and S30M50 are shown in Figure 7. The oxygen losses from room temperature to 800 °C are 0.13%, 0.13% and 0.20%, respectively, and their correspondent Δδ from room temperature to 800 °C are 0.022, 0.022 and 0.034. Besides, the loss (as the details shown in Figure A3) was nearly complete when the temperature was greater than 400 °C. S30M50 produced a greater oxygen vacancy than S30 and S30C4 at high temperatures (400–800 °C).

Table 4 summarizes the TGA results of S30, S30C4, S30M50 and LSCF6428. Clearly, LSCF6428 experienced much greater mass loss, implying the LSCF6428 generated greater oxygen vacancy. The ionic conductivity of LSCF6428 (ca. 1.0–1 × 10^−2^ S·cm^−1^ at 800 °C) [26,36] is higher than that (4 × 10^−3^ S·cm^−1^ at 800 °C) of the S30. Therefore, S50C20 was selected as the cathode to assemble SOFCs, and compared to the results from LSCF6428.

### 2.4. Performance of Cells

Standard half cells Ni+8YSZ/8YSZ/20SDC with different cathodes were made for comparison. One cell consisted of a porous cathode layer of 7–15 µm, a porous 5 µm SDC layer as a barrier, and a dense 15 µm electrolyte layer closely sintered on a 300 µm anode. The cathode was either made by S50C20, or LSCF6428 for comparison. The interfaces between the layers were intact. No cracks were observed.

**Figure 8:**
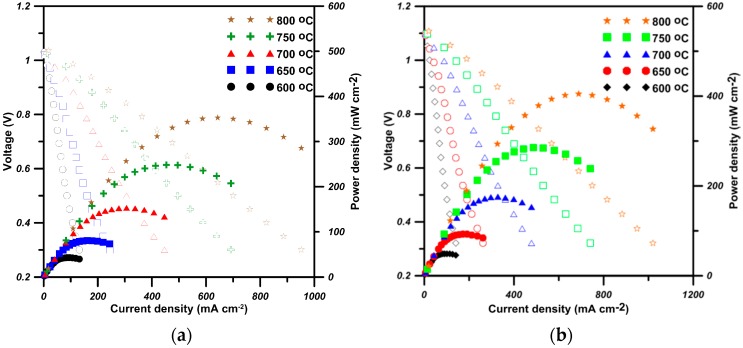
Output voltage and power density of the cells: (**a**) with the LSFC; and (**b**) with S50C20 cathode as a function of current density.

Figure 8 is the output voltage, current density and power density of the cells. The open circuit voltage (OCV) of the made cells show a value close to 1.1 V, implying the electrolyte is leakage-free. The maximal power density of the cells is 353 mW·cm^−2^ and 406 mW·cm^−2^, respectively, at 800 °C. The power density of the cell with S50C20 cathode is 15% higher than that of the cell consisting of LSCF.

## 3. Discussion

A substitution of trivalent cation (e.g., Bi^3+^) on the A-site of perovskite by divalent cation (e.g., Sr^2+^) induces two possible reactions in the materials. One is the increase in the valence state of B-site cations, and the other is the formation of oxygen vacancy. If the B-site cations are transition metals (M), the change in valence state creates M*^n^*^+^/M^(*n*+1)+^ couples, which act as the hopping sites for electrons via neighboring oxygen, as shown in Equation (2). A three-dimensional MO_6_ octahedral network [36] in the perovskite structure acts as the double exchange media and induces electronic conduction, causing valence exchange along the MO_6_ octahedron, as shown in Equation (2) [37]. The electrical conductivity of S30 (Figure 2a) and XPS results (Figure 5 and Figure 6) depicting the valence states of Fe (4^+^ and 3^+^) in nearly equal quantity at the B-sites are mostly favored as hopping sites (e.g., Fe^3+^/Fe ^4+^ and Mn^3+^/Mn^4+^) and generates electron conduction at low temperatures.

The ionic conductivity of the perovskite/fluorite structure occurs in more complex way at higher temperature via the vacancy mechanism of oxygen ion diffusion. Two factors, the concentration of the oxygen vacancy $\left[V_{\ddot{O}}\right]$ and the mobility µ_ο_ of the oxygen vacancy are effective and affect the oxygen ionic conductivity in the perovskite structure.

(4) $$\sigma_{i}=\mathrm{Ze}\;\left[V_{\ddot{O}}\right]{\;\mu }_{O}$$

However, there are several factors influencing the ionic mobility, basically, to which the ions block transport of the oxygen in the perovskite structure.

**Figure 9:**
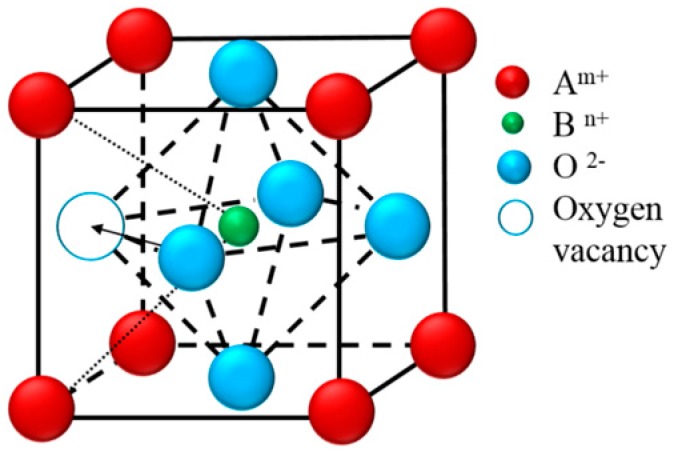
Schematic diagram of a perovskite structure and the triangle consisted of two A/one B atoms showing the vacancy-oxygen ion exchange vector perpendicular to the triangular plane.

Several structural factors have been proposed to explain the relationship of ionic transportation behavior with the oxygen vacancies, etc. These factors can be explained by a schematic diagram in Figure 9.

Goldschmidt et al. [38] defined a tolerance factor, *S*, to reveal the stability of the perovskite structure, as shown below:

(5) $$S=\frac{（r_{A}+r_{O}）}{\sqrt{2}（r_{B}+r_{O})}\;$$

where *r*_A_ and *r*_B_ are the mean ionic radii for A- and B-site cations, and *r*_O_ is the radius of an oxygen ion. For a stable perovskite structure, the tolerance factor *S* is between 0.77 and 1.0. When *S* = 1.0, a perfect cubic structure is achieved, and the B–O–B bond angle is 180°; when *S* < 1.0, the lattice structure tends to change from cubic to rhombohedral and to orthorhombic.

**Figure 10:**
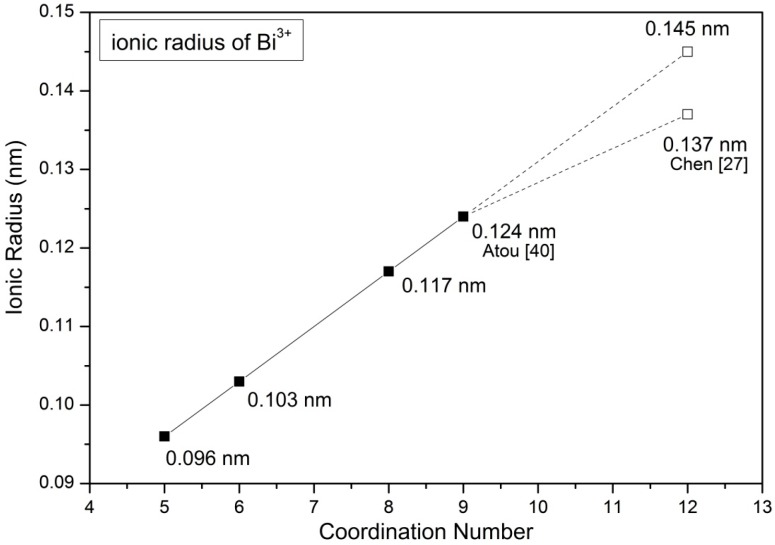
Ionic radius of Bi^3+^ with different coordination number (the solid points were obtained from Shannon [39] while the holly-square point is calculated from a linear relationship). Two data points (Atou [40] and Chen [27]) are plotted as well.

Table 5 shows the ionic radii of various cations of a specified coordination number (CN) referenced from Shannon [39]. Since the ionic radius of Bi^3+^ of CN = 12 is not reported in the literature, one extrapolated value was calculated from a linear relationship between the reported ionic radii and CNs, as shown in Figure 10. An ionic size 1.45 Å of Bi^3+^ is obtained, which is slightly greater than 1.37 Å calculated from X-ray data in our previous work [27].

If the value 1.45 Å of Bi^3+^ (CN = 12) was used to calculate the tolerance factor of LSCF6428, S30, S30N3 and S30M50, the results are shown in Table 6. Since the *S* values of all samples are less and close to 1.0, the result indicates all BSFM samples have a stable perovskite structure.

Other than the tolerance factor, Richter et al. [36] also proposed another parameter, critical radius *r*_cr_, to describe the open space of the triangle (Figure 9) allowing oxygen ion diffusion in the perovskite structure, and it could be calculated by the following equation:

(6) $$r_{\mathrm{cr}}=\frac{a_{0}\left(\frac{3}{4}a_{0}-\sqrt{2}r_{B}\right)+r_{B}^{2}-r_{A}^{2}}{2\left(r_{A}-r_{B}\right)+\sqrt{2}a_{0}}\;$$

where *a*_0_ is the pseudo-perovskite lattice parameter, and *a*_0_ can be derived experimentally or as below [41] if the structure is nearly cubic:

(7) $$a_{0}\approx V^{1/3}=2.37r_{B}+2.47-2.00\left(S^{-1}-1\right)$$

In other words, the *r*_cr_ describes the maximum size of the gap for a mobile oxygen ion to pass through the “saddle-point” between the triangle surround by two A-site cations and one B-site cation. The critical radii *r*_cr_ of four samples are calculated and shown in Table 5. As all critical radii are close to 0.90 Å and far smaller than the oxygen ions radius (1.40 Å in six-fold coordination or 1.36 Å in three-fold coordination in the saddle-point), the oxygen ions need a strong thermal vibration of the lattice to assist the migration [36].

Sammells et al. [42] introduced another parameter, lattice free volume *V*_f_, which was defined as equal to the difference in the unit cell volume (*V* = $a_{0}^{3})$ and the total volume of the constituent ions. Moreover, Hayashi et al. [43] modified the *V*_f_ and proposed the other parameter, the specific free volume (*V*_sf_) to describe the same diffusion phenomena:

(8) $$V_{\mathrm{sf}}=\frac{V_{f}}{V(\mathrm{unit}\_\mathrm{cell}\_\mathrm{volume})}$$

They used the parameters, *V*_f_ and *V*_sf_, as the bases to compare the free space in the perovskite structure of different compositions. The larger the *V*_sf_, the lower the driving force and activation energy of the migration. Based on the calculated results shown in Table 5, the difference between the two structural factors, the tolerance factor and critical radius are not significant, having less than 1% difference. However, a *V*_sf_ valued of S30 and S30M50 being 10% more than that of LSCF6428 provides more free space for mobile oxygen diffusing in the perovskite structure. Therefore, the structural advantage of S50C20 offering a lower activation energy (*E*_a_) of 1.2 eV and a higher mobility (µ_0_) of oxygen migration are expected. The power density output of the cell with the S50C20 cathode is better than that with LSCM6328.

## 4. Materials and Experimental Procedures

### 4.1. Synthesis of Cathode Materials

Bi_0.7_Sr_0.3_Fe_1−*x*_M*_x_*O_3−δ_ powders (M = Co, Ni, Mn) were synthesized by the EDTA-citric method [44]. Bi_2_O_3_ (Solar Applied Materials Technology Corp., Tainan City, Taiwan) was dissolved in a HNO_3_ solution (65%, Sigma-Aldrich, St. Louis, MO, USA), and Sr(NO_3_)_2_ (Showa Chemical Industry Co., Ltd., Tokyo, Japan), Fe(NO_3_)_3_·9H_2_O (Showa Chemical Industry Co., Ltd.), Ni(NO_3_)_2_·6H_2_O (Showa Chemical Industry Co., Ltd.), MnN_2_O_6_·4H_2_O (Alfa Aesar, Ward Hill, MA, USA) were separately dissolved in deionized (DI) water to obtain the solutions for a 0.1 M molar concentration. EDTA (ACROS, Geel, Belgium) was dissolved in the NH_4_OH solution (J.T. Baker Chemical, Center Valley, PA, USA) as a buffer solution. The nitrite solutions were mixed together as the formulation BSFM specified, then added drop-by-drop into the buffer solution, following the addition of citric acid (Showa Chemical Industry Co., Ltd.) in a molar ratio of EDTA/cations/citric acid = 1:1:2. After the solution was mixed and stirred, NH_4_OH was added to control pH to 7.0. Then the solution was heated on a hot plate at 200 °C until a gel solution was obtained. The dried gel was combusted at 200 °C in an oven and left overnight. The ash-like powder was obtained and calcined at 800 °C–900 °C to obtain a single-phase powder.

### 4.2. Preparation of Fuel Cell

SOFC was prepared by the following procedures. In brief, 8 mol % yttria stabilized zirconia (8YSZ, Tosoh Co., Tokyo, Japan), NiO (F grade, INCO Ltd., Toronto, ON, Canada), and mesophase carbon powder (MCMB, National Chung-Shan Institute of Science & Technology, Taoyuan, Taiwan) in a mass ratio of 45:45:10 were used to prepare three separated slurries in a solid loading of 28 vol %. The slurries contained de-ionized water as a liquid carrier and Darvan C (PMAA-N, Vanderbilt Co. Ltd., Norwalk, CT, USA) as dispersant. After ball-milling for 24 h, the slurries in a specified composition of the anode, were mixed for an additional 1 h, and then dried to powder form. The anode powder was pressed into the pellets of 20 mm in diameter and 1.0 mm in thickness. The pellets were calcined at 1000 °C for 2 h in air before spin-coating of the 8YSZ layer.

The slurry for spin-coating of 8YSZ electrolyte layer was prepared by mixing YSZ powder (TZ-8Y, Tosoh, Tokyo, Japan) with ethyl cellulose (E0266, TCI, Tokyo, Japan) and terpineol (regent grade, ACROS, NJ, USA) in a mass ratio of 30:2:68. The mixture was ball-milled for 24 h until the slurry was stable and homogeneous. Spin-coating of the YSZ slurry on the anode was conducted in three steps: (1) a few drops of the slurry were coated onto a spinning anode pellet in a turning rate increasing from 0 to 6000 rpm; (2) the spinning speed was kept at 6000 rpm for 60 s; and (3) after coating, the film and the substrate were co-calcined at 500 °C for 10 min. In some cases, the coating was repeated up to three times. A thick coating, made by three cycles, for example, was capable of forming a ca. 15 µm thickness 8YSZ electrolyte layer after sintering. Finally, the coated pellets (half cells) were sintered at 1400 °C for 4 h. A layer of cathode with the pastes of LSFC6482 was screen-printed on a half cell. The S50C20 cathode in an area of 1.00 cm diameter, and a thickness of ca. 10 µm was made. After drying and binder-burn-out of the printed cathode layer, the cell was sintered at 1000 °C for 2 h at a heating rate of 5 °C·min^−1^.

### 4.3. Characterization

**Figure 11:**
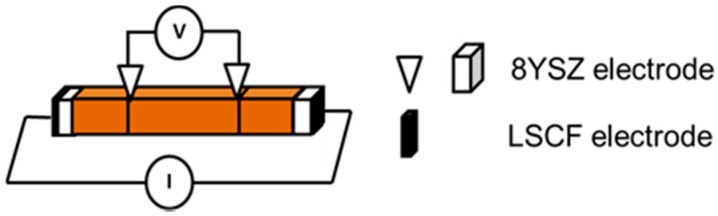
Schematic diagram of the four-probe DC method used for ionic conductivity measurement.

The crystal structure and phases identification of the calcined powder were determined by X-ray diffractometry (XRD, TTRAX 3, Rigaku Ltd., Tokyo, Japan). The total electrical conductivity of the sintered sample in a disk shape and the ionic conductivity of the rectangular samples were measured from 150 °C to 800 °C by a two-probe or four-probe DC technique (Figure 11), respectively [26]. The valence states of Fe and Mn in Bi_0.7_Sr_0.3_Fe_1−*x*_M*_x_*O_3−δ_ were investigated by X-ray photoelectron spectroscopy (XPS) (Thermo Scientific, Theta Probe, Waltham, MA, USA). Spectra were recorded before and after a step of bombardment cleaning using Ar^+^ ion at 3 keV for a selected period (0 s–180 s). Thermo-gravimetric analysis (TGA) was conducted for estimating the oxygen non-stoichiometry of the ferrite samples using a TA Q600 instrument (SDT Q600, TA Waters LLC, New Castle, DE, USA). The samples underwent through careful drying and sintering steps before the XPS and TGA tests.

## 5. Conclusions

Single-phase doped (Bi,Sr)FeO_3−δ_ materials were synthesized and displayed interesting mixed electronic and ionic conductivities at temperatures up to 800 °C. The electric conductivity of S*x*C*y* (*x* = 0–30, *y* = 0–5) shows two-stage temperature dependence, but the others (*x* = 40–50, *y* = 10–20) show only one-stage dependence. However, for the ionic conductivity of S*x* and S*x*C*y* samples, the dependence follows only one *E*_a_ 1.24 ± 0.06 eV to the entire temperature region 300–800 °C, which is lower than that (1.32–1.34 eV) of LSCF cathodes [11].

The solubility limit of Ni in S30 is 0.03, but there is no limit for Mn up to 0.50 to keep the single perovskite phase. Three samples, doped S30, S30N3 and S30M50, show CTE values ranging between 12.8 × 10^−6^ and 13.7 × 10^−6^ K^−1^, which are close to that of zirconia- or ceria-based electrolyte.

The results of the valence state of Fe by XPS show the fraction of Fe^3+^ and Fe^4+^ are in a ratio of 0.47 vs. 0.53 in S30M50 at room temperature. Similar XPS analysis on the Mn shows divalent and trivalent Mn ions appear in nearly equal quantity (0.46 vs. 0.44). Both B-site ions in equal quantity are favored for the double-exchange conduction of electrons along the Fe(Mn)O_6_ octahedral chains in the perovskite structure at lower temperature. At temperatures greater than 400 °C, the oxygen vacancies in BSF samples increase by more than a few percentages, and result in not only an interruption of the double-exchange mechanism, but also enhancement of a small-polaron conduction by the help in neighboring oxygen vacancies.

One of the structural factors, specific free volume (*V*_sf_) for BSFM materials, offers 10% more free space than LSCF6428 and may result in a better diffusive mobility of oxygen/vacancy in BSF materials at higher temperature.

All doped BSFs show similar two-stage temperature dependence of conductivity: when lower than the transition temperature (*T*_tr_), all samples show similar double-exchange conductive mechanism with an activation energy of 0.54–0.59 eV·mol^−1^ or 0.37–0.44 eV·mol^−1^. At temperatures higher than *T*_tr_, the conductive mechanism relies on a few percent concentration of oxygen vacancies, offering the exchange media to electrons to hop between small polarons ($e^{-}{:V}_{O}^{2+}$). This allows the cells with Co-doped S50C20 to reach a maximum power density of 406 mW·cm^−2^, with 15% being better than the cell with LSCF6428 testing at 800 °C.

## Figures and Tables

**Table 1:** Abbreviations and properties of Bi_1−*x*_Sr*_x_*FeO_3_ (*x* = 0.1 to 0.5) and Bi_0.5_Sr_0.5_Co*_y_*Fe_1−*y*_O_3_ (*y* = 0 to 0.2).

SrO-Doping (S*x*-Series)	Abbreviation	Sintering Temperature (°C) for 5 h	Sintered Density (% T.D.)	CTE
Bi_0.9_Sr_0.1_FeO_3_	S10	950	96.4	10.0
Bi_0.8_Sr_0.2_FeO_3_	S20	1000	95.9	11.5
Bi_0.7_Sr_0.3_FeO_3_	S30	1000	95.5	13.6
Bi_0.6_Sr_0.4_FeO_3_	S40	1050	95.3	14.3
Bi_0.5_Sr_0.5_FeO_3_	S50	1050	95.1	15.1
**SrO- and CoO-Doping (S*x*C*y*-Series)**	**Abbreviation**	**Sintering Temperature (°C) for 5 h**	**Sintered Density (% T.D.)**	**CTE**
Bi_0.5_Sr_0.5_Fe_0.95_ Co_0.05_O_3_	S50C5	1000	95.1	15.4
Bi_0.5_Sr_0.5_Fe_0.9_ Co_0.1_O_3_	S50C10	950	95.3	16.9
Bi_0.5_Sr_0.5_Fe_0.8_ Co_0.2_O_3_	S50C20	950	96.0	19.3

**Table 2:** Activation energy of electrical conductivity calculated from Arrhenius plots of electrical conductivity of Bi_1−*x*_Sr*_x_*FeO_3_ (*x* = 0.1 to 0.5) and Bi_0.5_Sr_0.5_Co*_y_*Fe_1−_*_y_*O_3_ (*y* = 0 to 0.2).

Composition	*E*_a_(HT) (eV)	*E*_a_(LT) (eV)	*T*_tr_ (°C)
S10	0.48	0.70	527
S20	0.27	0.65	523
S30	0.24	0.57	516
S40	0.22	0.48	506
S50	0.21	0.40	502
S50C5	0.20	0.38	497
S50C10	0.31	-	NA
S50C20	0.25	-	NA

**Table 3:** Activation energies at LT and HT, and the temperature of turning-point of doped S30.

Composition	*E*_a_(LT) (eV)	*E*_a_(HT) (eV)	*T*_tr_ (°C)
S30	0.57	0.24	516
S30N03	0.54	0.36	431
S30C4 [27]	0.59	0.25	496
S30M10	0.44	1.09	489
S30M50	0.37	1.09	396

Note LT: low temperature region; HT: high temperature region.

**Table 4:** Molecular weight, mass loss and oxygen non-stoichiometry (Δδ) of S30, S30C4, S30M50 and LSCF6428 at 800 °C.

Formula of the Doped S30 at 25 °C	*M*_sample_	*M*_oxygen_	Δ*m/m*_s_	Δδ
Bi_0.7_Sr_0.3_FeO_2.95_	275.617	16.0	0.13%	0.022
Bi_0.7_Sr_0.3_Fe_0.96_Co_0.04_O_2.97_	276.060	16.0	0.13%	0.022
Bi_0.7_Sr_0.3_Fe_0.5_Mn_0.5_O_2.88_	274.047	16.0	0.20%	0.034
La_0.4_Sr_0.4_Co_0.2_Fe_0.8_O_3−δ_	222.862	16.0	1.89%	0.26

**Table 5:** Ionic radii of cation used for calculation of the geometry factors [39].

Properties	A Site			B Site				O^2−^
	La^3+^	Sr^2+^	Bi^3+^	Mn^3+^ ^a^	Fe^3+^ ^a^	Co^3+^	Ni^3+^	
Ionic radius unit: Å	1.36	1.44	1.45 ^b^	0.645	0.645	0.545	0.56	1.40
			1.37 ^c^					
CN	12	12	12	6	6	6	6	6
Atomic mass	138.90	87.62	209.0	54.94	55.85	58.93	58.69	16.0

Note ^a^ High spin; ^b^ Calculated from Figure 10; ^c^ Referenced from Chen [27].

**Table 6:** Calculated tolerance factor, critical radius and specific free volume of LSCF6428, S30, S30N3, and S30M50 cathode materials.

Composition	Tolerance Factor, *S*	Critical Radius, *r*_cr_ (Å)	Specific Free Volume, *V*_sf_ (%)
La_0.6_Sr_0.4_Co_0.2_Fe_0.8_O_3_	0.975	0.910	20.1
Bi_0.7_Sr_0.3_FeO_3_	0.984	0.902	22.6
Bi_0.7_Sr_0.3_Fe_0.97_Ni_0.03_O_3_	0.986	0.901	22.5
Bi_0.7_Sr_0.3_Fe_0.5_Mn_0.5_O_3_	0.984	0.902	22.6
